# Unveiling the Synergistic Interaction Between Liposomal Amphotericin B and Colistin

**DOI:** 10.3389/fmicb.2016.01439

**Published:** 2016-09-13

**Authors:** Rita Teixeira-Santos, Elisabete Ricardo, Ricardo J. Branco, Maria M. Azevedo, Acácio G. Rodrigues, Cidália Pina-Vaz

**Affiliations:** ^1^Department of Microbiology, Faculty of Medicine, University of PortoPorto, Portugal; ^2^CINTESIS – Center for Research in Health Technologies and Information Systems, Faculty of Medicine, University of PortoPorto, Portugal; ^3^UCIBIO-REQUIMTE – Department of Chemistry, Faculty of Science and Technology, Universidade NOVA de LisboaLisboa, Portugal; ^4^Burn Unit, Department of Plastic and Reconstructive Surgery, Hospital São JoãoPorto, Portugal; ^5^Department of Microbiology, Hospital São JoãoPorto, Portugal

**Keywords:** liposomal amphotericin B, colistin, synergistic effect, *Candida* species, *Aspergillus fumigatus*

## Abstract

Patients with multiple comorbidities are often administered simultaneously or sequentially antifungals and antibacterial agents, without full knowledge of the consequences of drug interactions. Considering the clinical relevance of liposomal amphotericin B (L-AMB), the association between L-AMB and six antibacterial agents was evaluated against four clinical isolates and one type strain of *Candida* spp. and two clinical isolates and one type strain of *Aspergillus fumigatus.* In order to evaluate such combined effects, the minimal inhibitory concentration (MIC) of L-AMB was determined in the presence of 0.5-, 1-, 2-, and 4-fold peak plasma concentrations of each of the antibacterial drugs. Since the L-AMB/colistin (CST) association was the most synergic, viability assays were performed and the physiological status induced by this association was characterized. In addition, computational molecular dynamics studies were also performed in order to clarify the molecular interaction. The maximum synergistic effect with all antibacterial agents, except CST, was reached at fourfold the usual peak plasma concentrations, resulting in 2-to 8-fold L-AMB MIC reduction for *Candida* and 2-to 16-fold for *Aspergillus.* For CST, the greatest synergism was registered at peak plasma concentration (3 mg/L), with 4-to 8-fold L-AMB MIC reduction for *Candida* and 16-to 32-fold for *Aspergillus*. L-AMB at subinhibitory concentration (0.125 mg/L) combined with CST 3 mg/L resulted in: a decrease of fungal cell viability; an increase of cell membrane permeability; an increase of cellular metabolic activity soon after 1 h of exposure, which decreased until 24 h; and an increase of ROS production up to 24 h. From the molecular dynamics studies, AMB and CST molecules shown a propensity to form a stable molecular complex in solution, conferring a recognition and binding added value for membrane intercalation. Our results demonstrate that CST interacts synergistically with L-AMB, forming a stable complex, which promotes the fungicidal activity of L-AMB at low concentration.

## Introduction

Fungi are recognized as major pathogens in critically ill patients. *Candida* and *Aspergillus* species are the most common agents of invasive fungal infections (IFIs), although other yeasts and filamentous fungi are becoming emerging pathogens (Gullo, 2009). In the clinical practice, patients at risk for IFIs often receive concomitantly or sequentially antifungal therapy and antibacterial agents, either for prophylactic or therapeutic purposes (Stergiopoulou et al., 2009). However, this procedure is often adopted without full knowledge of the consequences resulting from pharmacological drug interactions.

A recent literature review showed that the combination of amphotericin B (AMB) or fluconazole with antibacterial agents that impair RNA or protein synthesis, such as rifampicin (RIF), azithromycin (AZM), clarithromycin (CLR), and tetracycline (TET), enhances the *in vitro* activity of the antifungal agent against yeast and filamentous fungi (Azevedo et al., 2015). In addition, Zeidler et al. (2013) demonstrated that colistin (CST), an antibiotic that targets Gram-negative membranes, exhibits a synergistic effect with antifungal agents belonging to the echinocandin class against *Candida* spp. According to the authors, the echinocandin weakens the fungal cell wall facilitating the colistin action upon fungal membranes, and, consequently, this effect enhances the antifungal activity of the echinocandin (Zeidler et al., 2013). These results are particularly promising since the available antifungal panoply is narrow, encompassing only a few classes of agents, and the discovery of new drugs is a slow and exhaustive process (Roemer and Boone, 2013). Moreover, some antifungals demonstrate limited efficacy, high toxicity, and are prone to the development of antifungal resistance. Therefore, the association of compounds that enhance the efficacy of antifungal drugs may contribute to a more effective reduction of fungal burden and minimize the development of resistance (Zeidler et al., 2013).

Although the association of antifungal and antibacterial agents may have beneficial implications in clinical terms, little is known about the pharmacological drug interactions and the underlying mechanisms of synergism. While there are few studies available addressing this topic, it is important to stress that antagonistic or indifferent effects may also be found (Petrou and Rogers, 1988; Stergiopoulou et al., 2009; Venturini et al., 2011). Thus, an extensive study aiming to evaluate the mechanisms involved in the association between antifungal and antibacterial agents upon fungal growth inhibition is mandatory.

We hereby propose to evaluate the association between liposomal amphotericin B (L-AMB) and several antibacterial drugs against *Candida* spp. and *Aspergillus fumigatus*, as well as to unveil the mechanisms underlying such drug interactions. In the case of synergistic interaction, we have investigated using molecular dynamics (MD) simulations whether (i) the antibacterial agent has an intrinsic activity upon fungal cells; or (ii) the antibacterial agent acts as a facilitator of AMB activity. MDs simulations have been widely applied to study the interaction of AMB molecules with the fungal membranes, since the mechanism of action of AMB is not well characterize (Baginski et al., 1997; Sternal et al., 2004; Czub and Baginski, 2006; Czub et al., 2007; Neumann et al., 2009; Cohen, 2010; Foglia et al., 2014). Thus, this approach may be essential to unveil the mechanism of synergism.

Our results demonstrate which antibacterial agents improve L-AMB efficacy and elucidate about the hypothetical underlying mechanism.

## Materials and Methods

### Strains and Growth Conditions

Four *Candida* clinical isolates and one type strain (C. *albicans* 596, *C. albicans* 38, *C. glabrata* 590, *C. krusei* 120, and *C. albicans* ATCC 90028) and two clinical isolates and one type strain of *A. fumigatus* (*A. fumigatus* 76, *A. fumigatus* 88, and *A. fumigatus* ATCC MYA-3626) were used in this study. Clinical isolates of *Candida* spp. and *A. fumigatus* were obtained from patients admitted at Centro Hospitalar São João, Porto, Portugal. Isolates are included in the Collection of fungal clinical isolates deposited at Department of Microbiology, Faculty of Medicine of Porto, Portugal. *Candida* isolates were grown in Sabouraud dextrose agar (SDA; Formedium, Norfolk, United Kingdom) at 35°C for 24 h (Teixeira-Santos et al., 2012); *Aspergillus* isolates were cultured in SDA at 35°C for 72 h (Faria-Ramos et al., 2014). *Escherichia coli* ATCC 25922 was also included in this study for control of antibacterial drugs.

### Antimicrobial Drugs and Susceptibility Testing

Liposomal amphotericin B (provided by Gilead Sciences, Inc, San Dimas, CA, USA), rifampicin (Sanofi Aventis, Anagni, Italy), azithromycin (Farmoz, Sintra, Portugal), clarithromycin (Alcala Farma, Madrid, Spain), colistin sulfate salt (C4461, Sigma–Aldrich, Munich, Germany), tetracycline (T3258, Sigma–Aldrich), and linezolid (LZD; Pfizer, New York, NY, USA), were prepared according to the manufacturer’s guidelines, in order to obtain stock solutions of 2 mg/L. The minimal inhibitory concentration (MIC) of L-AMB was determined according to M27-A3 protocol and M27-S4 supplement for *Candida* sp. and M38-A2 protocol of Clinical and Laboratory Standards Institute for *Aspergillus* sp. (CLSI, 2008a,b, 2012). The MIC of each antibacterial drugs was also determined; the tested antibacterial concentrations ranged between 0.25 and 128 mg/L. In order to evaluate the effect between L-AMB and the different antibacterial agents, the MIC to L-AMB was determined in the presence of 0.5-, 1-, 2-, and 4-fold peak plasma concentrations of each antibacterial drug. Peak plasma levels described in the literature are: RIF 12 mg/L (Van Ingen et al., 2011), AZM 4 mg/L (Sevillano et al., 2006), CLR 2 mg/L (Kees et al., 1995), CST 3 mg/L (Michalopoulos and Falagas, 2011), TET 2 mg/L (Agwuh and MacGowan, 2006), and LZD 12 mg/L (Dryden, 2011). Briefly, the drugs were diluted twofold in culture medium in order to obtain a 1:4 dilution. Fifty microliters of each L-AMB concentration (ranging from 0.03 to 16 mg/L) were combined with 50 μL of each of the concentration of the distinct antibacterial agents (described above). The plates were incubated at 35°C and the L-AMB MIC values determined after 24 and 48 h; MIC was the lowest concentration that prevented any discernible growth. Since clinical breakpoints were not yet established for L-AMB, the *Candida* isolates were classified in wild type (wt) whenever the MIC was ≤2 mg/L and non-wild type (nwt) when the MIC was >2 mg/L according to the epidemiological cutoff values (ECVs) proposed by Pfaller and Diekema (2012). For *A. fumigatus*, only the MIC value is displayed, since AMB ECVs and clinical breakpoints remain undefined (Pfaller and Diekema, 2012). *C. albicans* ATCC 90028 and *A. fumigatus* ATCC MYA-3626 type strains were used as controls, as recommended by CLSI protocol (CLSI, 2008a,b, 2012). *E. coli* ATCC 25922 was used as a bacterial quality control to assure that antibacterial dilutions were correct. MIC determination assays were performed in triplicate.

### Evaluation of Cell Viability

The evaluation of cell viability was performed with *C. albicans* 596, as a representative example, for the most synergic association, L-AMB/CST. The concentrations tested were selected according the susceptibility results. Yeast suspensions (10^6^ yeast cells/mL) were exposed to L-AMB 0.125 mg/L alone and in combination with CST 3 mg/L, for 24 h at 35°C, 150 rpm. Another suspension was prepared and treated with single CST 3 mg/L. At specific time points, 1, 3, 6, and 24 h, aliquots were collected and tested for viability. Cell viability was determined in triplicate by counting colony forming units (CFU). Briefly, the number of viable cells for each treatment was determined by plating 100 μL of serial dilutions on SDA agar medium and incubating at 35°C for 24 h; the number of CFUs was determined and compared with control plates (not exposed to drugs or exposed to each antimicrobial drug, L-AMB and CST); before being plated, cells were washed once and resuspended in fresh medium in order to prevent antifungal carryover.

### Functional Characterization of Drug Interaction

The distinct cellular physiological status resulting from the interaction between L-AMB and CST were assessed by flow cytometry in a time-course assay, using *C. albicans* 596 as a representative example. A cell suspension (10^6^ yeast cells/mL) was used in all assays described below. Yeast cells were incubated with single L-AMB 0.125 mg/L and in association with CST 3 mg/L during 24 h. In order to evaluate whether there was a CST concentration-dependent effect, the following treatment conditions were also tested: L-AMB 0.125 mg/L with (i) CST 1.5 mg/L and (ii) CST 6 mg/L. The cellular status induced by single CST treatment was evaluated as control. At specific time points, 1, 3, 6, and 24 h, aliquots were collected and evaluated. All cytometric evaluations were performed in a FACSCalibur cytometer (BD Biosciences, Sydney, NSW, Australia) standard model, equipped with three photomultipliers, standard filters, a 15-mW 488-nm Argon laser, and using CellQuest Pro software (version 4.0.2). All the assays were performed in triplicate.

The effect of L-AMB 0.125 mg/L alone and in association with CST 3 mg/L was evaluated regarding: (i) membrane potential, (ii) membrane integrity, (iii) metabolic activity, and (iv) endogenous reactive oxygen species (ROS) production.

#### Evaluation of Membrane Potential

The cell membrane potential was assessed by staining the cells with Bis-(1,3-Dibutylbarbituric Acid) Trimethine Oxonol (DiBAC_4_(3), Sigma–Aldrich), as described by Teixeira-Santos et al. (2012). The fluorescence intensity (FI) at FL1 (fluorescent detector; 530 nm) was registered and a staining index (SI) was defined as the ratio between the FI of treated cells and the FI of non-treated cells.

#### Evaluation of Membrane Integrity

Cell membrane integrity impairment was evaluated using propidium iodide (PI, Sigma–Aldrich) staining. After antimicrobial treatment, yeast cells were stained with 1 mg/L of PI for 30 min at 35°C, in the dark (Pina-Vaz et al., 2005). The FI was measured at FL3 (fluorescent detector; 630 nm). The amount of injured cells in each sample was defined as the percentage of PI-positive cells.

#### Evaluation of Metabolic Activity

Metabolic changes were evaluated using 5-Carboxyfluorescein diacetate (5-CFDA, Sigma–Aldrich), at 10 μM final concentration. Antimicrobial treated cells were stained with 5-CFDA and incubated for 45 min, at 35°C, at 150 rpm, in the dark (Liao et al., 2003). The mean intensity of fluorescence (MIF) was registered at FL1 (530 nm).

#### Evaluation of Endogenous ROS Production

Reactive oxygen species production was evaluated as previously described (Yan et al., 2009). In brief, yeast cells were incubated with 20 mg/L of 2′,7′-dichlorofluorescin diacetate (DCFH-DA, Sigma–Aldrich) for 30 min at 35°C, at 150 rpm. Cells were washed once (2,655 × *g* for 5 min at room temperature; 5417R, Eppendorf) and resuspended in phosphate-buffered saline (PBS, Sigma–Aldrich); afterward, cells were treated with the antimicrobials as described in “Functional characterization of drug interactions” section. FI was determined at FL1 (530 nm). ROS production was calculated by subtracting the FI value displayed by cells treated with antimicrobials from that of cells treated with both antimicrobials and DCFH-DA.

Simultaneously, 10 μL of the treated cell suspension were placed in a microscope slide for further analysis under fluorescence microscopy, in a Carl Zeiss Axiovert inverted microscope, using laser wavelength of 488 nm.

### Molecular Dynamics Studies

The Automated Topology Builder (ATB) web server was used for the AMB (C_47_H_73_NO_17_) and CST (C_52_H_98_N_16_O_13_) MDs topology parametrization, corresponding to the new residues name IDs D3JY and 09SS, respectively, as a GROMACS G54A7FF United-Atom force field (Malde et al., 2011).

The ATB derived force-field was tested in a series of independent MD simulations in an explicit water box. Then, AMB and CST molecules were placed in the same simulation box, together with 6230 SPCE water molecules, at more than 17 Å apart from each other, in order to avoid any computational bias. The entire MD protocol, including energy minimization, equilibration, and production steps, were performed using GROMACS 4.6.1 simulation package (Hess et al., 2008). MD simulations were performed at the in-house Fermi GPU high performance computing workstation. The global charge of the system was zero and simulations run under periodic boundary conditions in an isothermal-isobaric (NPT) ensemble at 300 K and 1.0 bar, during 20 ns, as described in previous studies (Branco et al., 2012).

### Statistical Analysis

Results are detailed as mean value and the respective standard deviation. Paired-sample Student’s *t-*test was used to compare the effect of L-AMB and CST alone and in association.

All statistical analysis was performed using the SPSS software (v. 23.0).

## Results

### Susceptibility to L-AMB alone and in Association with Antibacterial Drugs

Single RIF, AZM, CLR, CST, TET, and LZD did not inhibit the growth of all the fungal species studied, even at the maximum tested concentrations (fourfold peak plasma concentrations). The fungal strains showed variable susceptibility profiles to L-AMB, being all *Candida* strains wt (MIC value ≤ 1 mg/L), except *C. krusei* with a MIC value of 8 mg/L. Regarding *A. fumigatus* MIC values of 2 and 4 mg/L were registered (**Table 1**).

**Table 1:** Minimal inhibitory concentration (MIC) of liposomal amphotericin B alone and in association with several antibacterial drugs at fourfold peak plasma concentrations.

	MIC value of L-AMB (mg/L)					
Fungal isolates	L-AMB in association with					
	L-AMB	RIF	AZM	CLR	CST	TET	LZD
*C. albicans* ATCC 90028	1	0.25	0.25	0.25	0.125	0.25	1
*C. albicans* 596	1	0.125	0.25	0.25	0.125	0.25	1
*C. albicans* 38	0.5	0.06	0.25	0.25	0.06	0.25	0.5
*C. glabrata* 590	0.25	0.125	0.125	0.125	0.06	0.125	0.25
*C. krusei* 120	8	2	4	8	2	2	8
*A. fumigatus* ATCC MYA-3626	1	0.25	0.5	0.25	0.06	0.125	1
*A. fumigatus* 676	4	1	1	1	0.125	0.25	4
*A. fumigatus* 88	2	0.5	1	0.5	0.125	0.125	2

*L-AMB, liposomal amphotericin B; RIF, Rifampicin; AZM, Azithromycin; CLR, Clarithromycin; CST, Colistin; TET, Tetracycline; LZD, Linezolid.*

Minimal inhibitory concentration of L-AMB was reduced 2-to 4-fold in the presence of peak plasma concentrations of RIF, AZM, CLR, and TET. The maximum effect of these antibacterial agents was obtained at fourfold plasma concentrations with a reduction of L-AMB MIC of 2-to 8-fold, in the case of *Candida* (except CLR regarding *C. krusei* 120) and 2-to 16-fold for *A. fumigatus* (**Table 1**). CST at 0.5-fold peak plasma concentration (1.5 mg/L) resulted in a reduction of L-AMB MIC of 2-to 4-fold for all fungal isolates tested. At the peak plasma concentration (3 mg/L), CST reduced L-AMB MIC in 4-to 8-fold for *Candida* spp. and 16-to 32-fold for *A. fumigatus.* Interestingly, CST at 2- and 4-fold peak plasma concentration (6 and 12 mg/L, respectively) resulted in the same effect upon L-AMB MIC as CST 3 mg/L. Linezolid, even at fourfold peak plasma concentration did not associate with any L-AMB MIC reduction, regarding all tested fungal strains.

Colistin was selected for further studies since it was the drug that exhibited the highest synergistic effects with L-AMB.

### Impact of L-AMB/CST Association upon:

#### Fungal Cell Viability

**FIGURE 1:**
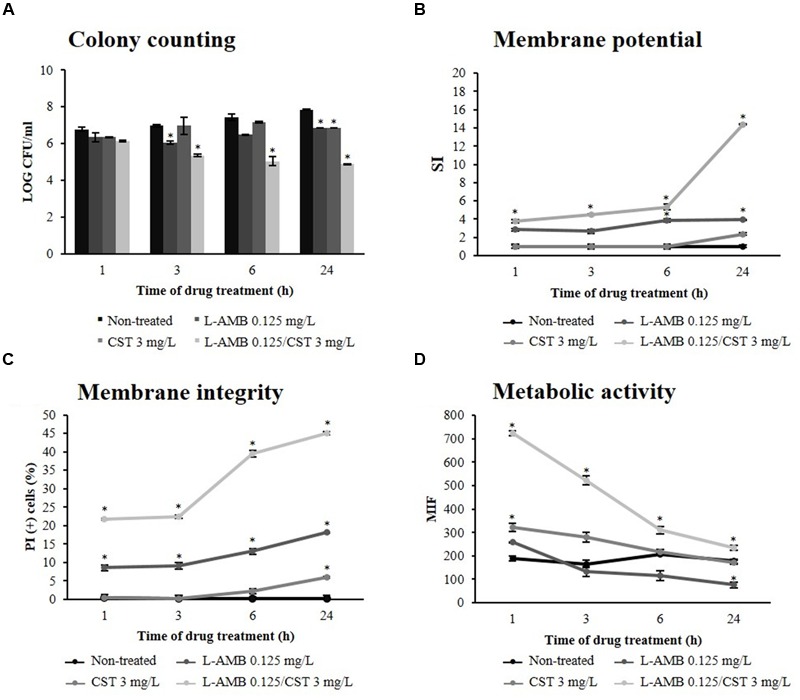
**Effect of liposomal amphotericin B (L-AMB) in combination with colistin upon *C. albicans* (strain 596) physiological parameters. (A)** Viability assessment by colony forming units (CFU) enumeration of *C. albicans* cells exposed to (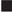) L-AMB, (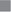) CST, and (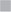) L-AMB in association with CST. The CFU counts of cells non-treated are represented as (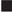). **(B)** Cell membrane potential was evaluated using DiBAC_4_(3) staining. **(C)** Cell membrane integrity was evaluated using propidium iodide (PI) staining. **(D)** Cell metabolic activity was evaluated using 5-CFDA staining. Data at respective time points corresponds to mean ± standard deviation. ^∗^*p*-values < 0.05, significant differences between the treatments with L-AMB alone and CST alone vs. the control (non-treated cells); and between the association L-AMB/CST and L-AMB alone.

**Figure 1A** represents *C. albicans* 596 viability at subinhibitory concentration of L-AMB (0.125 mg/L), alone and in association with CST 3 mg/L. Treatment with L-AMB 0.125 mg/L significantly decreased yeast cell viability up to 3 h of exposure (*p* = 0.039); however, after 3 h of treatment, cells recovered the ability to replicate and viability increased up to 24 h. At this time point, a significant difference was registered between cells treated with L-AMB and non-treated cells (*p* = 0.011). In the case of cells exposed to CST, significant growth reduction was only registered after 24 h of incubation (*p* = 0.009). Whenever cells were treated with L-AMB in association with CST there was a significant reduction of CFU counts (10^7^ to 10^5^ cells/mL) soon after 3 h of incubation (*p* = 0.037). This CFU reduction was consistently observed along the time up to 24 h (*p* = 0.004).

#### Cell Membrane Permeability

Liposomal amphotericin B acts on fungal cells by binding to the sterol component of the cell membrane, first leading to alterations in cell permeability (Gray et al., 2012). In order to evaluate whether antibacterial agents improve the effect of L-AMB on cell membrane permeability, the membrane potential and cell membrane integrity were assessed with the fluorescent dyes DiBAC_4_(3) and PI, respectively. DiBAC_4_(3) enters only in depolarized cells, where it binds reversibly to intracellular components, resulting in an increased fluorescent signal. The results obtained regarding membrane depolarization of *C. albicans* 596 are depicted in **Figure 1B**; after 3 h of treatment, the SI of cells exposed to L-AMB 0.125 mg/L was 2.65 (*p* = 0.173); at 6 h, the SI increased to 3.88 (*p* = 0.043), remaining constant up to 24 h (*p* = 0.042). Whenever yeast cells were exposed to CST, the SI was about 1.00 up to 6 h of treatment (*p* = 0.082), increasing to 2.36 at 24 h (*p* = 0.052). Regarding the association of L-AMB 0.125 mg/L with CST 3 mg/L, soon after 1 h of treatment the SI was 3.79, increasing up to 14.38 following 24 h. Significant differences were observed between the association L-AMB/CST and single L-AMB at 1 h (*p* = 0.025), 3 h (*p* = 0.028), 6 h (*p* = 0.011), and 24 h (*p* = 0.011). L-AMB 0.125 mg/L in association with CST 1.5 mg/L induced an increase of SI up to 24 h (SI = 6.69; *p* = 0.028). This effect was more pronounced when L-AMB 0.125 mg/L was combined with CST 3 mg/L, as previously described. However, when L-AMB 0.125 mg/L was combined with CST 6 mg/L no significant differences were found, compared to the association L-AMB/CST (3 mg/L; data not shown).

Such results show the key role of L-AMB/CST association on the increase of cell membrane depolarization.

Conversely, PI is a cell viability marker, which enters cells only when membrane has been seriously injured. The results regarding membrane lesion of *C. albicans* 596 are represented in **Figure 1C**. In cells treated with L-AMB 0.125 mg/L, the % of PI(+) cells after 1 h of exposure was about 10.00%, increasing slightly up to 24 h (% of PI(+) cells = 14.93); significant differences were found at 1 h (*p* = 0.019), 3 h (*p* = 0.036), 6 h (*p* = 0.008), and 24 h (*p* = 0.036), compared to control (non-treated cells). Cells treated with COL 3 mg/L showed PI-uptake only after 24 h of incubation, with a % of PI(+) cells equal to 5.93 (*p* = 0.046). Concerning the L-AMB/CST association, soon after 1 h of exposure, PI stains 21.82% of the treated cells, and after 6 h of incubation, this value reaches 39.47%; at 24 h, the percentage of cells with membrane lesion was about 45.01%. Significant differences were observed between the L-AMB/CST association and L-AMB treatment at 1 h (*p* = 0.011), 3 h (*p* = 0.008), 6 h (*p* = 0.002), and 24 h (*p* = 0.040). Thus, data suggests that L-AMB/CST association promotes the membrane lesion effect of L-AMB, in a time-dependent fashion.

#### Metabolic Activity

To assess the metabolic effects of L-AMB/CST upon *C. albicans* 596, the cells were stained with 5-CFDA; 5-CFDA is a cell-permeant esterase substrate that measures enzymatic activity (Breeuwer et al., 1995; Boender et al., 2011). Only the cells metabolically active will display a high level of fluorescence. The results obtained for MIF are detailed in **Figure 1D**. MIF displayed by viable cells (not exposed to the drugs) remained constant up to 24 h; at this time point, the MIF was 176.99. Cells treated with L-AMB did not display significant differences in MIF up to 3 h (*p* = 0.198). After 6 h, MIF decreased over time up to 75.53 (*p* = 0.049) at 24 h of exposure. When yeast cells were treated only with CST 3 mg/L, the MIF increased after 1 h (322.59; *p* = 0.029), indicating that the cells were metabolically active. However, after 3 h of treatment, the MIF decreased up to a value of 172.11 (*p* = 0.220), at 24 h, similar to the MIF of cells not exposed to the drugs. Cells exposed to the association L-AMB/CST initially displayed a very high MIF (724.30); however, it decreased until 24 h of treatment (234.91). Significant differences were registered between the metabolic activity of cells exposed to the drug association and to L-AMB alone at 1 h (*p* = 0.001), 3 h (*p* = 0.021), 6 h (*p* = 0.014), and 24 h (*p* = 0.025).

#### Endogenous ROS Production

**FIGURE 2:**
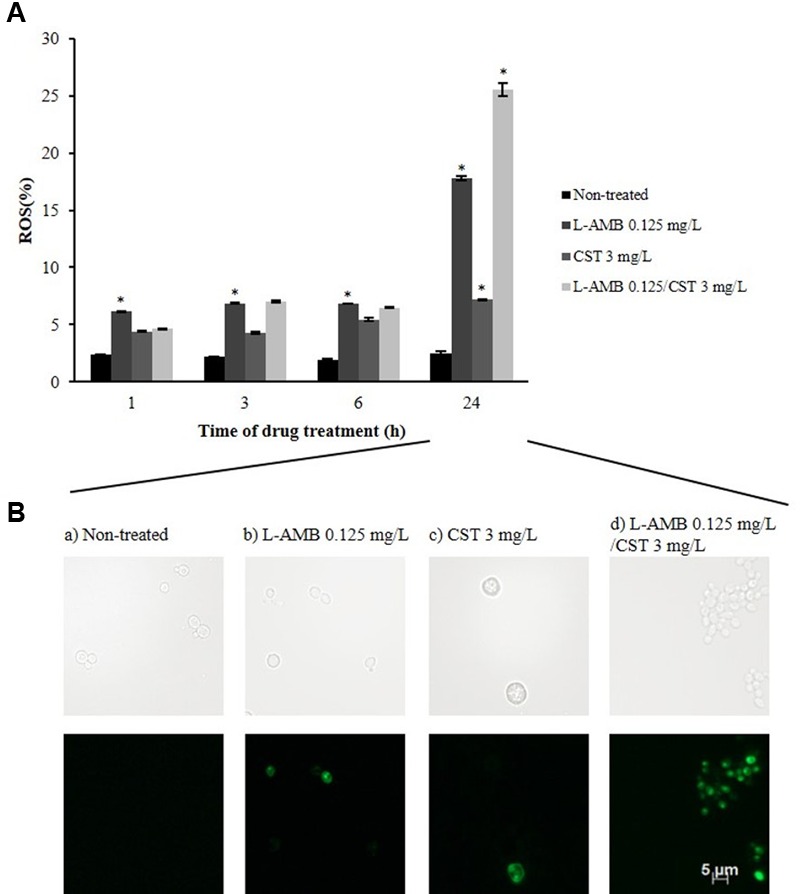
**Effect of L-AMB in association with colistin upon endogenous ROS production. (A)** Endogenous reactive oxygen species (ROS) production determined by DCFH-DA staining, assessed by flow cytometry. **(B)** Fluorescence microscopy imaging showing ROS-positive cells after treatment with (b) L-AMB and (c) CST alone and (d) in association after 24 h of exposure; (a) control, non-treated cells. ^∗^*p*-values < 0.05, significant differences between the treatments with L-AMB alone and CST alone vs. the control (non-treated cells); and between the association L-AMB/CST and L-AMB alone.

Reactive oxygen species production was assessed by DCFH-DA staining. DCFH-DA is oxidized to highly fluorescent 2′,7′-dichlorodihydrofluorescein (DCF) by ROS. ROS production by cells exposed to L-AMB 0.125 mg/L was constant up to 6 h of incubation (≈7.00%; *p* = 0.005); at 24 h, the number of fluorescent cells increased up to 18% (*p* = 0.001) (**Figure 2A**). Exposure to CST 3 mg/L resulted in reduced ROS formation in *C. albicans* 596 cells, reaching a value of 7.00% (*p* = 0.010), following a 24 h of incubation. Images obtained by fluorescence microscopy (**Figure 2B**) showed vesicle formation in the presence of CST, after 24 h of incubation. Cells exposed to L-AMB/CST displayed a growing ROS production pattern; 7.00% following 6 h (*p* = 0.056); 25.53% following 24 h of incubation (*p* = 0.022) (**Figure 2A**). These results reveal that L-AMB/CST leads to an increase of intracellular accumulation of ROS in relation to single L-AMB after 24 h.

### Self-Association Propensity Assessed by Atomic Molecular Dynamics Simulations

**FIGURE 3:**
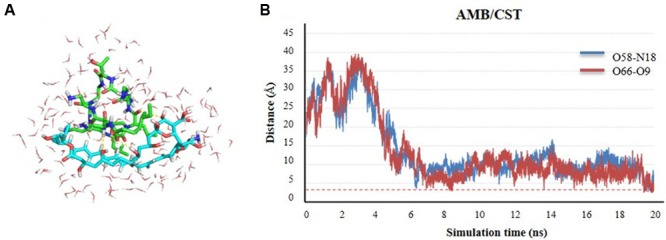
**Self-assembly of amphotericin B (AMB) and colistin (CST; represented in sticks) in water medium (represented in lines) through molecular dynamics (MD) simulation studies. (A)** Complex conformation snapshot after 20 ns of simulation, including water molecules within 4 Å of the complex. **(B)** Time evolution of the h-bond distance between two molecules during the complex formation.

It is known the propensity of AMB to form dimeric or even higher complex tetrameric structures once in water medium, which interfere with the electrophysiology of living cells through transmembrane ion channel formation (Starzyk et al., 2014). Herein, we describe the propensity of AMB and CST in water also to self-assembly based on MDs simulations. The results demonstrate that the two molecules tend spontaneously form a natural complex in solution, starting from a perfect unbound, solvated form, separated by more than 17 Å. Once the two molecules meet each other after the first 4 ns of simulation, they are steadily attracted into a complex formation that stands stable for the rest of the simulation time. This complex is characterized by polar interactions, namely hydrogen bond interactions between the C_5_- and C_9_-OH groups of AMB and amide groups of CST, as depicted in **Figures 3A,B**. These two H-bond pairs (AMB-O_58_:CST-N_18_ and AMB-O_66_:CST-O_9_) converged for a minimum distance of 2.7 and 2.5 Å, respectively. It seems that the complex is quite stable on the simulation window, supporting an analogous mechanism to the one predicted and validated experimentally for the dimerization of AMB alone. However, the main dimer-stabilizing contacts of the AMB:CST system seems to be the polar interactions and not the van der Waals forces contribution, as described for the hydrophobic nature of dimerization process of AMB molecules, which still might be essential for speeding up transmembrane intercalation of the complex and consequent disruption of the cell membrane through pore formation, resulting into a perfect and synergetic Trojan mechanism for the antimicrobial internalization (Starzyk et al., 2014).

## Discussion

This study demonstrates that all the tested antibacterial agents except linezolid interact synergistically with L-AMB against several pathogenic fungi, namely *C. albicans, C. glabrata, C. krusei*, and *A. fumigatus* (**Table 1**). These antibacterial agents alone did not exhibit antifungal activity, thus suggesting that such compounds do not intrinsically target the fungal cells. The agents tested belong to distinct antibacterial classes with different mechanisms of action. Rifampicin inhibits the DNA-dependent RNA polymerase (RNAP), leading to the suppression of RNA synthesis and cell death (Campbell et al., 2001). The basic architecture of bacterial RNAP and yeast RNAP presents several structural differences (Minakhin et al., 2001), explaining why RIF by itself does not display antifungal activity. Azithromycin and CLR are macrolide antibiotics that bind to 50S ribosomal subunits of bacteria, blocking the protein synthesis and inhibiting cell growth (Kanoh and Rubin, 2010; Parnham et al., 2014); LZD also binds to the 50S subunit of the prokaryotic ribosome, blocking the assembly of a functional initiation complex for protein synthesis (Livermore, 2003); TET inhibits protein translation in bacteria by binding to the 30S ribosomal subunit (Chopra and Roberts, 2001). Although the basic mechanism of protein synthesis in eukaryotes is similar to that in bacteria, some noteworthy differences were described between the structure of eukaryotic and prokaryotic ribosomes (Berg et al., 2002). Colistin belongs to the family of polymyxin antibiotics, which target bacterial cell membrane. CST displaces Mg^2+^ and Ca^2+^ ions, which stabilize the negatively charged lipopolysaccharide LPS, disrupting the membrane integrity in gram-negative bacteria (Falagas and Kasiakou, 2005). The antifungal activity of polymyxin antibiotics against several fungal species has been previously reported (Schwartz et al., 1972); however, at the hereby tested concentrations, CST did not inhibit fungal growth. Colistin plasma concentrations usually range from 0 to 15 mg/L, with free CST levels ranging from 0.5 to 3 mg/L (Akers et al., 2015). It is important to emphasize that about 50% of CST molecules bind to plasma proteins (Falagas and Kasiakou, 2005).

Considering all the antibiotic drug combinations evaluated, the one that resulted in the strongest synergic effect was L-AMB/CST. Additionally, the synergistic combination of CST with other antifungal agents, namely echinocandins class, has been previously described (Zeidler et al., 2013). Therefore, we explored the underlying mechanism of synergism of L-AMB/CST interaction.

Amphotericin B is a lifesaving antifungal drug used to treat deep-seated fungal infections, exhibiting broad-spectrum fungicidal activity. This activity is based on its interactions with fungal cell membranes (Starzyk et al., 2014). AMB primarily binds to ergosterol, inserts into the cytoplasmic membrane, and forms pore-like structures; the result is osmotic instability, loss of membrane integrity, metabolic disruption, and ultimately cell death (Gray et al., 2012). Our results showed a significant growth reduction of fungal cells treated with L-AMB/CST during 24 h of exposure (10^8^ to 10^5^ cells/mL), with a simultaneous increase of cell membrane permeability, as documented by DiBAC_4_(3) (SI was 14.38) and PI staining (% of PI(+) cells was 45.01%) (**Figures 1B,C**). Altogether, these results strongly indicate that the association of CST significantly improves the fungicidal activity of L-AMB.

It is known that yeast cells exposed to L-AMB pressure reprogram their metabolism in response to an environmental stress (Zhang et al., 2002; Belenky et al., 2013; Teixeira-Santos et al., 2015). The combination of L-AMB and CST triggered an increase of metabolic activity in treated cells, soon after 1 h of exposure (3.8-fold higher than single L-AMB treatment; **Figure 1D**), suggesting an early strong stress response induced by membrane permeabilization. Along 24 h of exposure, the metabolic activity decreased overtime, nevertheless, still exhibiting a MIF 3.1-fold higher than single L-AMB treatment (**Figure 1D**). This decrease can be explained by membrane pore formation, which may cause the accelerated loss of fluorescence, or by a reduced metabolic activity (Breeuwer et al., 1995; Teixeira-Santos et al., 2015). Interestingly, the higher cell metabolic activity induced by the association of L-AMB/CST versus single L-AMB along 24 h period can be related with a higher endogenous ROS production in cells exposed to this association (**Figure 2**), which will result in oxidative damage and possibly is involved in induced programmed cell death (Phillips et al., 2003; Al-Dhaheri and Douglas, 2010). Curiously, single CST (3 mg/L) has a significant effect on ROS production by itself. Accordingly, cells exposed to CST display a different phenotype compared to cells exposed to the other treatment conditions, i.e., the formation of vesicles (apoptotic bodies), which is a characteristic event of apoptosis (Fink and Cookson, 2005). Although the functional studies that support the mechanism of action of L-AMB/CST association have been conducted only in *C. albicans*, according to the MIC determination assays, this mechanism seems to be transversal to the different species studied.

All of our results point to a considerable improvement of L-AMB antifungal effect, at subinhibitory concentrations, whenever associated to CST 3 mg/L. However, how do AMB and CST molecules interact? The computational molecular dynamics results demonstrate that the two molecules spontaneously form a natural complex in solution, characterized by a strong bond 1:1. Thus, AMB and CST molecules act together on fungal cells. In support of such a finding was the fact that the maximum synergistic effect was detected in the presence of peak plasma concentration of CST (3 mg/L); its increase to fourfold peak plasma concentration did not increase the synergistic effect, as documented by MIC determination and membrane potential evaluation assays. Moreover, a recent study described that the interactions of AMB with biomembranes are managed by the molecular organization of AMB (Starzyk et al., 2014). AMB self-associates to dimeric structures; AMB dimers can further assemble into tetramers, which induce the formation of transmembrane ion channels, impairing the electrophysiological homeostasis of a living cell (Starzyk et al., 2014; Davis et al., 2015). Considering all these findings, it is possible that CST binds to AMB molecules and accelerates the assembly of AMB tetramers, thus inducing pore formation on fungal cell membranes, also triggering a strong cell stress response, which is typical of AMB action.

Our results are extremely promising since AMB is an important therapeutic alternative for the treatment of IFIs, particularly when infection persists, despite treatment with other drugs, and the clinical response to AMB is reduced in about 40% of treated patients (Mora-Duarte et al., 2002; Ito and Hooshmand-Rad, 2005; Park et al., 2006). Simultaneously, this synergic association may be a clue for drug discovery.

Studies are being conducted in order to characterize the functional groups of the AMB/CST complex that interact with the fungal membrane aiming to design a more active antifungal compound.

## Author Contributions

RT-S, AR, and CP-V contributed with the experimental design and results interpretation of this study. RT-S, ER, MA carried out all experiments and RB perform the molecular dynamics studies. RT-S and CP-V wrote the manuscript and all authors performed a critical revision and approved the final version.

## Conflict of Interest Statement

The authors declare that the research was conducted in the absence of any commercial or financial relationships that could be construed as a potential conflict of interest.
